# Varying Properties of Extracellular Matrix Grafts Impact Their Durability and Cell Attachment and Proliferation in an *In Vitro* Chronic Wound Model

**DOI:** 10.1155/2024/6632276

**Published:** 2024-04-26

**Authors:** Katrina A. Harmon, Miranda D. Burnette, Justin T. Avery, Kelly A. Kimmerling, Katie C. Mowry

**Affiliations:** Organogenesis, 2 Perimeter Park South, Suite 310E, Birmingham, AL 35243, USA

## Abstract

While acute wounds typically progress through the phases of wound healing, chronic wounds often stall in the inflammatory phase due to elevated levels of matrix metalloproteinases (MMPs) and proinflammatory cytokines. Dysregulated expression of MMPs can result in the breakdown of extracellular matrix (ECM) formed during the wound healing process, resulting in stalled wounds. Native collagen-based wound dressings offer a potential wound management option to sequester excess MMPs and support cellular interactions that allow wound progression through the natural healing process. Herein, we utilized commercially available ECM matrices, two derived from porcine small intestinal submucosa (PCMP, 2 layers; PCMP-XT, 5 layers) and one derived from propria submucosa (ovine forestomach matrix, OFM, 1 layer), to demonstrate the impact of processing methodologies (e.g., layering and crosslinking) on functional characteristics needed for the management of chronic wounds. Grafts were evaluated for structural composition using scanning electron microscopy and histology, ability to reduce MMPs using fluorometric assays, and durability in an *in vitro* degradation chronic wound model. Both intact (nondegraded) and partially degraded grafts were assessed for their ability to serve as a functional cell scaffold using primary human fibroblasts. Grafts differed in matrix substructure and composition. While all grafts demonstrated attenuation of MMP activity, PCMP and PCMP-XT showed larger reductions of MMP levels. OFM rapidly degraded in the *in vitro* degradation model (<3 hours), while PCMP and PCMP-XT were significantly more durable (>7 days). The ability of PCMP and PCMP-XT to serve as scaffolds for cellular attachment was not impacted by degradation *in vitro*. Three ECM grafts with varying structural and functional characteristics exhibited differential durability when degraded in a simulated chronic wound model. Those that withstood rapid degradation maintained their ability to function as a scaffold to support attachment and proliferation of fibroblasts, a cell type important for wound healing.

## 1. Introduction

With over 10% of the US population affected by diabetes, diabetic foot ulcers (DFUs) are a common complication with a lifetime incidence of 19–34% and a high rate of recurrence, estimated at around 40% within 1 year and 65% within 5 years [1]. The risk of infection and amputation is increased the longer a DFU remains open [2]. Patients who undergo a lower extremity amputation face a 5 year mortality rate of more than 50% [3]. Treatment with advanced treatment modalities, primarily including extracellular matrix (ECM) grafts derived from human placental membranes and animal tissue, results in statistically fewer amputations, emergency department visits, and readmissions compared to those not treated with advanced therapies [4].

While acute wounds progress through the hemostatic, inflammatory, proliferative, and remodeling phases of healing, dysregulation in any phase can lead to stalled healing, resulting in a chronic wound [5, 6]. The microenvironment of chronic wounds is distinct from acute wounds and is often characterized by a prolonged period in the inflammatory phase with high levels of matrix metalloproteinases (MMPs) and other proteolytic enzymes promoting excessive ECM degradation that also influences cell signaling through release of factors sequestered in the ECM [7, 8]. While careful regulation of MMPs is critical to balance ECM deposition and degradation in normally healing wounds, the chronic wound microenvironment is highly aggressive; clinically, biopsies from DFUs harbored 65- and 2-fold increased concentrations of MMP-1 and MMP-8 compared to nondiabetic acute wounds [9]. MMP-1 (collagenase I) and MMP-8 (collagenase II) are known to play a major role in the increased ECM destruction characteristic of chronic wounds [7, 8].

One promising treatment for chronic wounds is the use of ECM grafts. They primarily consist of the structural proteins of native tissues that provide a scaffold for cells while maintaining properties that allow the graft to support cellular proliferation, migration, and homeostasis [4, 10–12]. Two examples of ECM scaffolds for wound management are small intestinal submucosa (SIS) and ovine forestomach matrix (OFM). SIS was first used in the 1960s as a vascular substitute [13]. Since then, the utility of SIS has been demonstrated in various reconstructive therapies including wound repair, hernia repair, colon/rectal surgery, bladder repairs, and musculoskeletal reconstruction [14]. OFM has emerged relatively recently as an ECM graft material, with the first 510K clearance obtained in 2010. Documented use of OFM includes covering a tissue deficit, reinforcing soft tissue, and supporting repair of damaged tissue [15]. Limited published studies are available around the applications of OFM; however, extensive characterization of the product has demonstrated that OFM contains the key components of an ECM graft [16]. Despite these successful applications, limitations of animal derived materials have been identified, including a relatively high level of variability in the starting material due to natural variation in tissue [14]. These limitations are common with tissue-derived products and are addressable through careful implementation of manufacturing controls.

One benefit of ECM grafts is their modulation of aberrant MMP activity by serving as a sacrificial substrate to quench the enzyme activity. While matrices derived from denatured collagen are generally soluble and easily absorbed [17], native collagen ECM grafts preserve the inherent protein structure providing a more durable framework with properties that enable enhanced functionality as a scaffold for cell adhesion and migration [18, 19]. ECM grafts have been engineered with a variety of beneficial properties, including coating with antimicrobial agents (e.g., polyhexamethylene biguanide (PHMB) and ionic silver), functionalizing with other molecules, or crosslinking or layering to confer increased stability and resistance to degradation [20–24].

Here, the physical and functional properties of three commercially available native ECM grafts were evaluated to assess the impact of processing methodologies (e.g., layering and crosslinking) on matrix structure and protease reduction; furthermore, an *in vitro* degradation chronic wound model was developed to determine scaffold degradation dynamics and functionality. Two grafts of crosslinked native type I collagen ECM derived from porcine SIS embedded with antimicrobial PHMB (PCMP, 2 layers, and PCMP-XT, 5 layers) were compared to a non-crosslinked OFM (1 layer), derived from the ECM of propria submucosa from ovine forestomach tissue [16]. This work demonstrates the impact of graft composition and processing on durability and functionality in its role as a protective barrier and scaffold to support wound healing.

## 2. Materials and Methods

### 2.1. Extracellular Matrix Scaffolds

Three native collagen ECM grafts, two derived from porcine SIS and one derived from ovine propria submucosa, were tested in this study. PCMP (2-layer; PuraPly® AM, Organogenesis) and PCMP-XT (5-layer; PuraPly® -XT, Organogenesis), which consist of layered, 1-ethyl-3-(3-dimethylaminopropyl)carbodiimide hydrochloride (EDC) crosslinked native type I collagen ECM derived from SIS embedded with antimicrobial PHMB, and OFM (1-layer; Endoform, Aroa Biosurgery), which consists of non-crosslinked native ECM derived from the propria submucosa isolated from ovine forestomach tissue, were compared. Of note, while all grafts contain perforations, PCMP-XT contains larger and higher frequency of perforations than either PCMP or OFM.

### 2.2. Scanning Electron Microscopy

The structure and surface characteristics of ECM grafts were assessed using scanning electron microscopy (SEM; Quanta 650 FEG, FEI; Hitachi SU3500; Hitachi High-Tech America Inc.). Samples were cut to the size of the SEM mount, mounted using conductive tape, and assessed directly without manipulation. Images were captured using top-down and cross-sectional views.

### 2.3. Histological Assessment

Histological samples were fixed in 4% paraformaldehyde and paraffin embedded. Serial sections 5 *μ*m thick were cut from the tissue blocks, placed onto charged glass slides (Super-Frost Plus, Fisher Scientific), and dried overnight at 60°C. Sections were deparaffinized, rehydrated, and stained using standard regressive hematoxylin and eosin (H&E) techniques [25]. Sections were then mounted with a cover slip and images were captured on an inverted microscope (Nikon Eclipse Ti, Nikon; EVOS M5000, Thermo Fisher Scientific).

### 2.4. Quantification of ECM Graft Thickness

H&E images of tissues were captured on a Nikon DS-Fi2 at 4x magnification (0.85 *μ*m/pixel). Images were stitched together, and the resulting graft image was divided into 20 equal regions along the length of the tissue. A random number generator was used to determine which of the 20 regions were to be measured (up to 9 per sample). The thickness was measured in triplicate per region using ImageJ (National Institute of Health); resulting measurements were averaged across regions to report the thickness per graft. For each graft type, two representative stitched images were assessed for three independent lots of material.

### 2.5. MMP Activity Assay

Fluorometric Drug Discovery Kits (Enzo Life Science) were used to assess the ability of OFM, PCMP, and PCMP-XT to reduce activity of a wide range of MMPs. Two independent assays were conducted as previously described [26] with minor modifications described herein. Briefly, 6 mm biopsy punches (*n* = 6–9 measurements per graft) were hydrated with phosphate buffered saline (PBS) and excess PBS was blotted off. Biopsy punches were added to 90 *μ*L of MMP (MMP-1 (15.3 Units), -2 (1.17 Units), -3 (2.00 Units), -8 (1.84 Units), -9 (0.89 Units), -10 (1.00 Units), -13 (1.38 Units), and -14 (2.40 Units)) buffers and were incubated at 37°C for 1 hour. Supernatants were then transferred to a white 96-well microtiter plate. The residual enzymatic activity was quantified with the addition of 10 *μ*L of the fluorogenic substrate, and fluorescence was measured every minute for 10 minutes on a BioTeK Synergy H1 plate reader at 328/420 excitation/emission (Agilent Technologies). The reaction velocity was calculated by plotting the relative fluorescence units against time to calculate the percent of MMP inhibited.

### 2.6. *In Vitro* Degradation Wound Models

OFM, PCMP, and PCMP-XT were first treated (*n* = 3 per graft type per run, 3 independent runs) with collagenase type I (310 U/mL; Worthington BioChem) or collagenase type II (230 U/mL; Worthington BioChem) in PBS. Additionally, an *in vitro* degradation model consisting of simulated wound fluid (SWF) [27] was adapted herein with the addition of collagenases type I and II (SWF+) to more closely model a chronic wound environment. Samples were incubated for up to 7 days at 37°C with gentle rocking. Every two to three days, the supernatant was collected and replaced with fresh solution. At days 3 and 7, samples were briefly rinsed with deionized water and dried overnight at 60°C. Dry samples were weighed and the percentage of tissue remaining was calculated. To qualitatively assess the impact of *in vitro* SWF+ degradation on scaffold structure, dried samples were fixed after 3 or 7 days of exposure to SWF+ and evaluated for histological changes using H&E staining per methods outlined above.

### 2.7. Assessment of Collagen throughout Degradation Time Course

PCMP and PCMP-XT scaffolds exposed to SWF+ were analyzed for relative concentrations of soluble and insoluble collagen (*n* = 3 per graft type per timepoint). Day 0, 3, and 7 samples were assayed per manufacturer instructions using the Sircol Soluble Collagen Assay and Sircol Insoluble Collagen Assays (BioColor). Briefly, dried scaffolds were minced and extracted overnight at 4°C in 0.1 mg/mL pepsin in 0.5 M acetic acid. Supernatants were utilized in the Sircol Soluble Collagen Assay while remaining solids were extracted in the fragmentation reagent included in the kit before use in the Sircol Insoluble Collagen Assay. Soluble and insoluble collagen concentrations were measured using a BioTek Synergy H1 plate reader (Agilent Technologies). Remaining solids were collected and dried overnight at 60°C prior to taking dry weights to determine the percentage extracted relative to the initial degradation weight.

### 2.8. Assessment of Mechanical Properties

To investigate changes in mechanical properties after exposure to a simulated wound environment, tensile testing was conducted on dehydrated samples (*n* = 8–10 per graft type at each timepoint) of nondegraded (day 0) or PCMP and PCMP-XT incubated in SWF+ for 3 days. Due to complete OFM degradation within 3 hours, samples from this group were excluded. Prior to tensile testing, matrix thickness of each sample was measured using calipers to enable calculation of Ultimate Tensile Strength (UTS) and Young's Modulus. Samples were cut into 1.25 cm × 5 cm rectangles and placed in pneumatic grips 2 cm apart. Using a 50 N load cell, a displacement rate of 25 mm/min was applied and the amount of displacement and force applied for each tissue was determined on an Instron Tensile Model 3342 (Instron).

### 2.9. Assessment of Fibroblast Attachment and Growth

Two independent lots of primary human fibroblasts (Lonza, Basel, Switzerland; 19TL196110 were donated from a 66-year-old white male, 22TL346465 from a 45-year-old white female) were thawed, expanded per manufacturer instructions, and used in the two cell seeding methods (static and dynamic) outlined below.

For the static seeding model, intact scaffolds were cut using 10 mm biopsy punches and placed into ultra-low attachment 24-well plates with a stainless-steel washer (6 mm inner diameter) on top to hold the tissue in place. Scaffolds (PCMP or PCMP-XT) were hydrated with 1 mL of growth medium (GM) consisting of Dulbecco's Modified Eagle Medium (DMEM) + 10% fetal bovine serum (FBS) for 10–30 minutes. GM was aspirated post rehydration, and a 20 *μ*L suspension of 50,000 fibroblasts in GM (8,333 cells/cm^2^ of washer inner diameter) was seeded onto grafts before being incubated at 37°C and 5% CO_2_ for 4 hours to allow for initial attachment. After initial attachment, 1 mL of GM was added to each well and incubated overnight. One day after seeding, all grafts were gently moved to a new ultra-low attachment plate with 1 mL of GM, which was changed every 2-3 days.

For the dynamic seeding model, scaffolds were cut using 6 mm biopsy punches and placed into 0.6 mL microcentrifuge tubes with 40,000 fibroblasts in 0.4 mL of GM (6,667 cells/cm^2^). Tubes were placed into a tube rack then incubated under gentle rocking at 37°C and 5% CO_2_. After 4 hours, grafts and media were moved to ultra-low attachment 24-well plates. The following day, all grafts were gently moved to a new ultra-low attachment plate and 1 mL of GM was added and subsequently changed every 2-3 days.

At days 1, 3, and 7, grafts seeded using either static or dynamic culture were assayed using an AlamarBlue metabolic assay (*n* = 3 per graft type per cell lot; ThermoFisher), as described in detail elsewhere [28]. Briefly, GM was exchanged for 10% AlamarBlue working solution in assay media (DMEM + 2.5% FBS) for 3 hours under standard culture conditions. Following incubation, fluorescence of the supernatant was measured in duplicate using a plate reader (BioTek Synergy H1; Agilent Technologies). Reduction of AlamarBlue was calculated and the number of cells on each scaffold was determined by generating a standard curve based on cells seeded at known densities. Immediately following the assessment, AlamarBlue solution was replaced with 1 mL GM. To qualitatively assess cell growth, day 3 and 7 dynamic seeded grafts were stained with Calcein AM (Invitrogen) per manufacturer instructions and imaged on an inverted microscope (EVOS M5000, ThermoFisher).

To assess cell attachment throughout the degradation process, 3- and 5-day SWF+ degraded grafts (*n* = 3 per graft type per cell lot) were evaluated using the static culture model and samples were assayed using AlamarBlue after 24 hours of attachment as described above. To qualitatively assess attachment, samples were fixed in 4% paraformaldehyde for at least 24 hours prior to staining for immunofluorescence. Briefly, grafts were rinsed with PBS, permeabilized with 0.25% Triton X-100/PBS for 45 minutes, and then blocked overnight at 4°C in 5% BSA/PBS with gentle rocking. After overnight blocking, samples were rinsed and stained with 4′,6-diamidino-2-phenylindole (DAPI, Invitrogen) for 20 minutes and then briefly rinsed for 10 minutes prior to imaging (Nikon Eclipse Ti, Nikon).

### 2.10. Statistical Analysis

All statistical analysis was completed using GraphPad Prism (GraphPad Software). A one-way or two-way analysis of variance (ANOVA) with Tukey's or Sidak's multiple comparison test was conducted. For all graphs, average ± standard deviation is reported. *P* values <0.05 were considered statistically significant. For degradation studies, best fit linear regressions and associated statistics were conducted to calculate and compare differences in slopes with a constraint of 100% dry weight at 0 days of exposure.

## 3. Results

### 3.1. Structural Characterization of ECM Scaffolds

**Figure 1:**
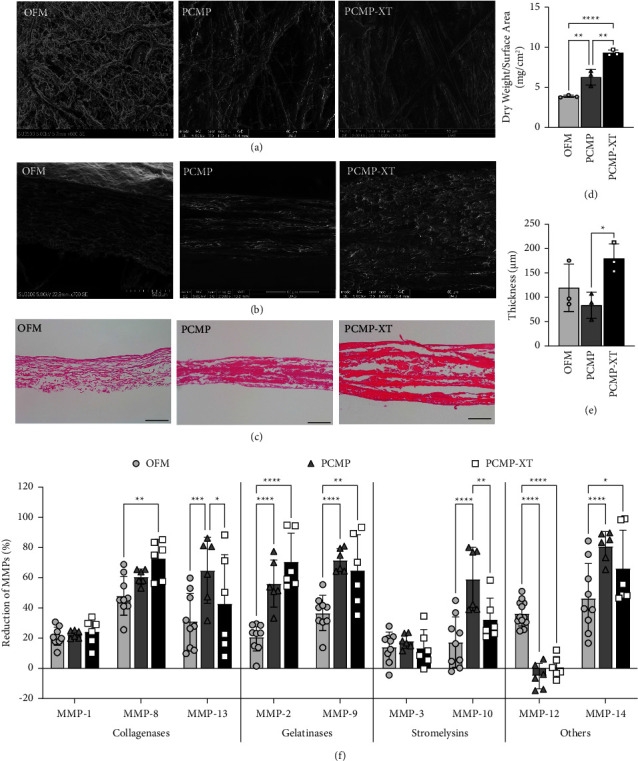
Characterization of extracellular matrix scaffolds. Representative scanning electron microscopy images of (a) top-down and (b) cross-sectional views of OFM, PCMP, and PCMP-XT. (c) Representative cross-sectional images (20x) of H&E-stained matrices. (d) Average dry weight per surface area and (e) tissue thickness; bars represent average ± standard deviation; markers represent individual average measurements from 3 grafts. (f) Reduction of MMP activity by ECM scaffolds. Bars represent average ± standard deviation; markers represent individual measurements (*n* = 6–9 measurements per graft; 3 grafts total). Asterisks denote significance at ^*∗*^*p* < 0.05, ^*∗∗*^*p* < 0.01, ^*∗∗∗*^*p* < 0.001, or ^*∗∗∗∗*^*p* < 0.0001. Scale bar indicates 50 *μ*m for all images.

SEM imaging revealed a more fibrous structure in OFM compared to PCMP or PCMP-XT, which were similar and more tightly packed (Figures 1(a) and 1(b)). These findings were consistent with histological observations, where PCMP and PCMP-XT demonstrated denser ECM staining compared to OFM, and the dry weight per surface area, where OFM was the lightest and PCMP-XT the heaviest (Figures 1(c) and 1(d)). When comparing tissue thickness based on H&E images, PCMP-XT was found to be significantly thicker than PCMP, while PCMP and OFM were comparable (Figure 1(e)).

### 3.2. ECM Scaffolds Diminished MMP Activity

OFM, PCMP, and PCMP-XT all decreased the activity of MMPs, with varying efficacy against collagenases (MMP-1, -8, and -13), gelatinases (MMP-2 and -9), stromelysins (MMP-3 and -10), and other MMPs (MMP-12 and -14) (Figure 1(f)). In general, PCMP and PCMP-XT resulted in more robust diminution than OFM; of the 9 targets tested, only MMP-12 activity was more significantly decreased by OFM compared to PCMP and PCMP-XT. PCMP-XT did not demonstrate a greater reduction of MMP activity compared to PCMP; instead, PCMP reduced significantly more MMP-13 and MMP-10 activity than PCMP-XT. Both MMP-8 (collagenase II) and MMP-14 activity were diminished by more than 40% for all three grafts, with PCMP and PCMP-XT diminishing activity by more than 60% for both targets. MMP-1 (collagenase I) and MMP-3 activities were similarly attenuated by all three grafts.

### 3.3. ECM Scaffolds Differentially Degrade In Vitro

**Figure 2:**
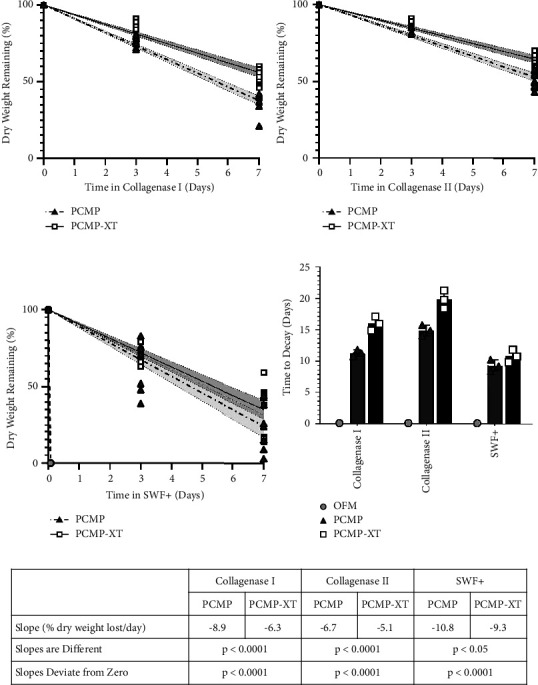
Time-course of *in vitro* degradation of ECM scaffolds in (a) collagenase type I, (b) collagenase type II, and (c) simulated chronic wound model with collagenase types I and II (SWF+). Points represent *n* = 3 measurements for each graft; lines and shading represent best fit linear regressions and 95% confidence intervals. (d) Predicted time to decay based on best fit linear regression for PCMP and PCMP-XT. Error bars represent 95% CI. (e) Summary of linear regression analyses.

ECM scaffolds were subjected to enzymatic degradation with collagenase type I (Figure 2(a)) or type II (Figure 2(b)) in PBS or in a SWF+ model including both collagenases to simulate a chronic wound environment (Figure 2(c)). OFM degraded rapidly, with complete loss of the ECM scaffold within 3 hours in all three models, and was therefore omitted from further analysis of scaffold functionality. Both PCMP and PCMP-XT demonstrated statistically significant degradation from starting weight in all three fluids, with PCMP demonstrating a statistically significant increased degradation rate compared to PCMP-XT in all three fluids (Figures 2(d) and 2(e)). PCMP and PCMP-XT scaffolds degraded most rapidly in the SWF+ model and most slowly in collagenase II. While PCMP and PCMP-XT remained intact through the experimental endpoint of day 7, based on the best fit linear regressions and confidence intervals, scaffolds are predicted to decay within 9–15 days for PCMP and 11–20 days for PCMP-XT in the three degradation models. In the most aggressive model with SWF+, PCMP is expected to resorb in 8–10 days, while PCMP-XT is expected to resorb within 10–12 days.

### 3.4. Maintenance of Scaffold Properties throughout Degradation

**Figure 3:**
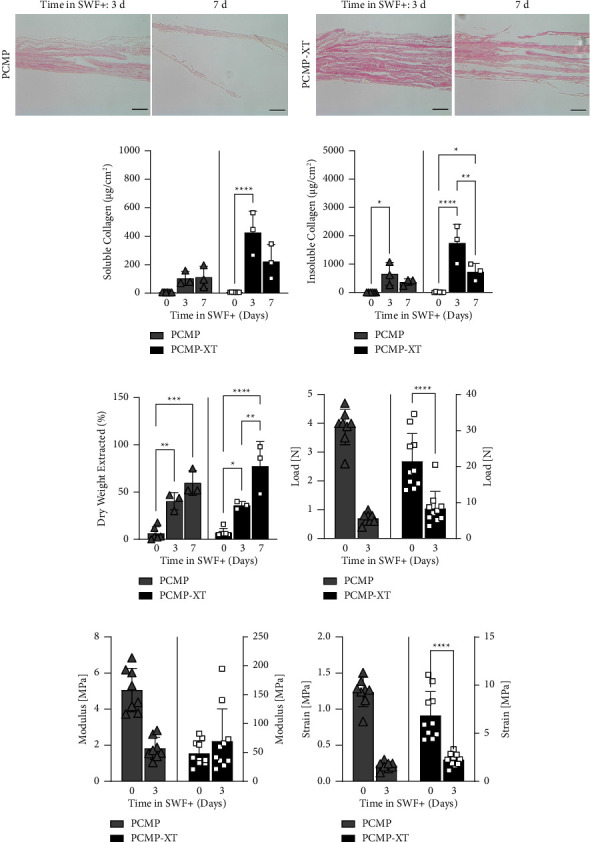
Characterization of PCMP and PCMP-XT after exposure to chronic wound model (SWF+). (a, b) Histological assessment of PCMP and PCMP-XT after 3 and 7 days in SWF+. 20x magnification shown; scale bar indicates 50 *μ*m. (c) Soluble and (d) insoluble collagen content of PCMP and PCMP-XT after 3 and 7 days in SWF+ and (e) extraction efficiency. Mechanical properties of intact and 3-day degraded PCMP and PCMP-XT: (f) load, (g) modulus, and (h) strain. Bars represent average ± standard deviation; points represent individual measurements (*n* = 3 per graft for collagen content; *n* = 8–10 for mechanical testing). Asterisks denote significance at ^*∗*^*p* < 0.05, ^*∗∗*^*p* < 0.01, ^*∗∗∗*^*p* < 0.001, and ^*∗∗∗∗*^*p* < 0.0001.

The ECM structure of PCMP and PCMP-XT during degradation in the SWF+ model was evaluated using H&E (Figures 3(a) and 3(b)). After 3 days of exposure to SWF+, PCMP and PCMP-XT appeared relatively intact with similar structural characteristics to nondegraded samples (Figure 1(c)). By day 7, both scaffolds demonstrated substantially less ECM staining with more void space. Overall, PCMP-XT matrices had a higher maintenance of ECM integrity throughout the 7 day degradation period compared to PCMP.

The collagen makeup of ECM scaffolds was analyzed following extraction in acetic acid. Collagen quantification in intact matrices was relatively low, which is likely attributable to low extraction efficiency from crosslinked matrices. Consistent with that hypothesis, *in vitro* degradation of both PCMP and PCMP-XT in SWF+ resulted in an increase in both soluble (Figure 3(c)) and insoluble (Figure 3(d)) collagen content extracted at day 3—likely due to improved extraction efficiency as crosslinked collagen was released from the matrix—which was statistically significant for all but the PCMP soluble content group. By day 7, the impact of degradation appeared to overcome the increased extraction efficiency, where collagen content in both PCMP and PCMP-XT trended downward for all groups except soluble collagen in PCMP. This decrease was only significant for the PCMP-XT group for insoluble collagen, which likely had the largest assay window with the highest levels of both collagen content and crosslinking due to the composition including 5 layers. To further assess the potential impact of extraction efficiency on quantified collagen content, the remaining solid matrices were dried and weighed post-extraction and normalized to the initial degradation weight (Figure 3(e)). As expected, degradation resulted in a strong increase in extraction efficiency at day 3 for both PCMP and PCMP-XT which was further increased in PCMP-XT at day 7. Increased extraction efficiency was observed for PCMP at day 7, though not statistically significant.

Finally, intact and 3-day degraded PCMP and PCMP-XT were subjected to mechanical testing (Figures 3(f)–3(h)) to assess maintenance of ECM structure. Seven-day degraded grafts were not analyzed due to technical challenges associated with handling the degraded matrices. For both intact and degraded conditions, PCMP-XT was significantly stronger with a higher elastic modulus than PCMP—consistent with the expectation of additional layers conferring additional strength and stiffness to the material. While the modulus was unchanged with degradation of either PCMP or PCMP-XT, the maximum load and strain of PCMP-XT were both significantly decreased after degradation in the *in vitro* chronic wound model, but of note remained stronger than PCMP. Similar trends, though not statistically significant, were observed for PCMP.

### 3.5. Fibroblast Attachment and Proliferation on ECM Scaffolds

**Figure 4:**
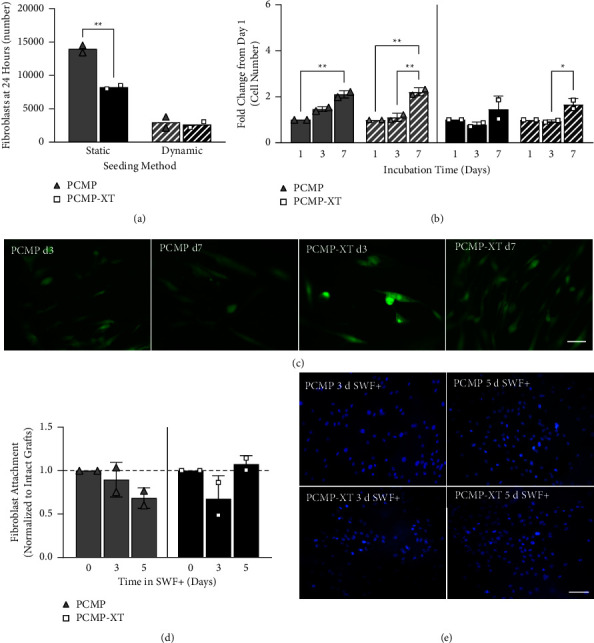
Scaffold properties of PCMP and PCMP-XT intact and after exposure to the *in vitro* chronic wound model. “Initial cell attachment (a) and increasing cell number over time (b) with static and dynamic seeding of fibroblasts on intact ECM matrices. (c) Representative immunofluorescence (Calcein AM) of fibroblasts attached to scaffolds 3 and 7 days after dynamic seeding. (d) Fibroblast attachment 24 hours after static seeding onto intact, 3-day, and 5-day degraded PCMP and PCMP-XT. (e) Representative DAPI-stained (blue) images of fibroblast attachment to degraded matrices. Bars represent average ± standard deviation; points represent average result of three replicates from *n* = 2 independent cell lots. Asterisks denote significance at ^*∗*^*p* < 0.05, ^*∗∗*^*p* < 0.01, ^*∗∗∗*^*p* < 0.001, or ^*∗∗∗∗*^*p* < 0.0001. 20x magnification shown; scale bar indicates 50 *μ*m; solid bars = static seeding and hashed bars = dynamic seeding.

Having determined that PCMP and PCMP-XT are maintained throughout 7 days of exposure to the *in vitro* chronic wound model, the ability of both intact and degraded grafts to serve as a scaffolding to support cell attachment and growth was evaluated using primary human fibroblasts (Figure 4). Intact grafts were evaluated for cell attachment and proliferation over 7 days, while grafts that underwent 3 or 5 days of degradation were tested for their capacity to support cell attachment over 24 hours in static cultures. Fibroblasts readily attached to both PCMP and PCMP-XT, with PCMP demonstrating statistically more attachment compared to PCMP-XT on day 1 (Figure 4(a)). Decreased attachment on PCMP-XT is likely a function of the static seeding method, due to the highly perforated nature of PCMP-XT compared to PCMP. To address this limitation, PCMP and PCMP-XT were also assayed using a dynamic cell seeding model. In this model, cell attachment to PCMP-XT and PCMP was comparable, supporting the hypothesis that increased perforations on PCMP-XT contributed to the differences observed in the static model (Figure 4(a)). PCMP-attached cells demonstrated proliferation, which was statistically significant in the dynamic model by day 3 and in both models by day 7, whereas on PCMP-XT, cells were maintained at day 3 and by day 7 demonstrated proliferation that was statistically significant in the dynamic model (Figure 4(b)). Cell attachment on dynamically seeded grafts was visualized on days 3 and 7 using Calcein AM; while cells were localized primarily to the surface on day 3 (particularly for PCMP-XT), hallmarks of cell invasion including increased elongation and cell spindles were observed on both grafts by day 7 (Figure 4(c)).

To further assess the ability of ECM scaffolds to support attachment and growth of cells, grafts that underwent 3- or 5-day degradation in SWF+ were assessed for their ability to support fibroblast attachment. Both PCMP and PCMP-XT demonstrated robust attachment of fibroblasts on 3 and 5 day degraded samples, which was unchanged from attachment to their nondegraded (day 0) counterparts (Figures 4(d) and 4(e)). These data provide further evidence that not only do PCMP and PCMP-XT function as scaffolds for cells important for wound repair, but they also maintain that ability as they naturally degrade.

## 4. Discussion

To build on the current evidence base and further elucidate how processing methodologies (e.g., layering and crosslinking) impact key properties, we utilized commercially available products constructed of SIS and OFM and evaluated matrix structure and protease attenuation; this analysis was complimented by the development and application of an *in vitro* degradation chronic wound model to study scaffold degradation dynamics and functionality [4, 29–31]. Here, both SIS and OFM were able to attenuate MMPs; however, structural and functional properties determined the magnitude of MMP reduction. Additionally, differing structural and functional properties resulted in varying durability. ECM grafts that were crosslinked resisted rapid degradation and were found to function throughout their degradation as a scaffold to support fibroblast attachment, growth, and invasion.

Normal wound healing progresses through four phases: the hemostatic phase, the inflammatory phase, the proliferative phase, and the remodeling phase [32]. However, chronic wounds fail to progress through the normal wound healing process, often stalling in the inflammatory phase for a number of reasons, including excessive bacteria/biofilm, increased neutrophil and proinflammatory (M1) macrophage accumulation, increases in proinflammatory cytokines and MMPs, and decreases in tissue inhibitors of matrix metalloproteinases [6, 33–35]. Collagen matrices and ECM grafts have been shown to support chronic, nonhealing wounds' progress through the wound healing cascade through several mechanisms, including cell attachment, structural support, and support of angiogenesis [17]. In this study, two classes of ECM grafts were assessed: SIS, which has been leveraged in tissue engineering applications since the 1970s, and OFM, which more recently emerged. The products in this study are both native collagen ECMs, with SIS undergoing layering and an additional chemical crosslinking process. Both tissue sources have been shown clinically to support progression through the wound healing phases [29, 30].

One of the defining characteristics of chronic wounds is an imbalance in MMP levels [36]. Aberrant expressions of collagenases (MMP-1, -8, and -13) and gelatinases (MMP-2 and -9) are hallmarks of the chronic wound microenvironment, with MMP-8 levels often being highly elevated due to continual neutrophil recruitment in the inflammatory state [37]. OFM and SIS both have been previously shown to inhibit protease activity [26, 38]. In this study, both OFM and SIS-based products (PCMP/PCMP-XT) decreased the activity of a wide range of MMPs. PCMP and PCMP-XT were both found to have a statistically greater impact on collagenases, gelatinases, and stromelysins than OFM; only MMP-12 was more significantly attenuated by OFM. Interestingly, MMP-12 has been shown to act as a positive regulator of epithelial cell proliferation and epidermal cell spreading in wounds [39].

In addition to reduction of proteolytic activity, an important property of ECM-based grafts in the context of a chronic wound environment is their ability to withstand rapid degradation. Various studies have modeled matrix integrity and longevity in a SWF comprised of salts with bovine albumin; however, these SWF environments likely underestimate a chronic wound environment, which is often characterized by high levels of inflammatory and catabolic proteins [27, 40]. *In vitro* enzymatic studies have been used to investigate the mechanisms of degradation, including assessing the kinetics and interaction of MMPs with collagen-based dressings [41–43]. Here, SWF was supplemented with collagenases I and II to generate a degradative chronic wound model mimicking protease levels found in wound exudate clinically [7, 8, 44]. PCMP and PCMP-XT exhibited resistance to *in vitro* enzymatic degradation in collagenase type I or II alone and within the harsher SWF+ model, whereas OFM was highly susceptible to degradation and completely degraded within 3 hours in all three solutions. Resistance to degradation is likely due to the chemical crosslinking of PCMP and PCMP-XT matrices, which is consistent with other studies demonstrating benefits of chemically crosslinking collagen matrices for tuning degradation time and persistence [41, 44, 45]. Despite demonstrating comparable reduction of collagenases I and II to PCMP, PCMP-XT was physically more resistant to degradation in SWF+, likely due to additional layers (5 versus 2) conferring additional durability—though not in a linear fashion.

Furthermore, studies have demonstrated that various cell types can attach and proliferate on SIS and OFM grafts [16, 18, 26, 38]. In this study, two seeding methods (static and dynamic) were utilized to evaluate human dermal fibroblast attachment, a key cell type in the wound healing cascade. PCMP and PCMP-XT supported cell attachment in both seeding methods. While PCMP demonstrated more attachment in the static seeding model compared to PCMP-XT, these differences were not observed with dynamic seeding, suggesting that the decreased attachment on PCMP-XT in the static model was likely due to increased perforations complicating the direct cell seeding onto the graft. However, the differences could also be attributable to increased matrix rigidity and thickness compared to PCMP; indeed, others have demonstrated the importance of substrate elasticity on cell attachment and motility [46]. Scaffold functionality was also examined following degradation of PCMP and PCMP-XT for 3 and 5 days in SWF+; degradation did not impact the ability of PCMP and PCMP-XT to support fibroblast attachment. Due to the rapid degradation of OFM observed in the *in vitro* wound model, OFM was not evaluated in this study for scaffold functionality. However, previously published literature has demonstrated that nondegraded OFM can support fibroblast migration, differentiation, and infiltration [16].

There are some limitations associated with this *in vitro* study. PCMP and PCMP-XT, which are embedded with antimicrobial PHMB, were compared to OFM without an antimicrobial, despite the availability of an antimicrobial version of OFM with 0.3% ionic silver. While the use of the antimicrobial OFM may represent a more direct comparison, antimicrobial activity was not a component of this study, and any potential cytotoxic effects of ionic silver would have obscured comparisons [47, 48]. Another limitation of this study was performing mechanical testing on dehydrated grafts, which prohibited a direct comparison to previously published data on hydrated OFM [49]. In this study, since mechanical testing was only utilized as an indicator of matrix integrity during degradation, this is minimally impactful; observed trends in the relative strength of grafts would be expected to be similar regardless of hydration status.

Chronic wounds often stall in the inflammatory phase, necessitating advanced modalities to support their progression through the wound healing cascade. In DFUs, the most common chronic wound, the use of advanced ECM modalities has shown significant benefit to patients by supporting wound healing [29, 30] while reducing amputations, emergency department visits, and readmissions [4]. ECM grafts have been leveraged for wound care applications due to their three-dimensional ECM and biocompatibility, as well as their ability to tune important properties including strength, porosity, and stability [31]. The use of advanced treatment modalities, such as biologically derived ECM scaffolds, can support the progression of chronic wounds through the phases of wound healing via several mechanisms including providing a moist wound healing environment, attenuating excess proteases, and functioning as a scaffold to facilitate cell attachment and growth. This study leveraged a novel *in vitro* degradation chronic wound model to assess matrix characteristics and their impact on stability over time. Crosslinking and layering appeared to play a major role in the durability and maintenance of ECM structure and functionality throughout degradation.

## 5. Conclusions

The role of ECM grafts in supporting wound healing has been established; however, the practicality of using these grafts in a clinical environment largely hinges on their ability to function while withstanding the harsh chronic wound microenvironment. Matrix durability can depend on several physical and functional properties; native ECM grafts derived from biological tissues offer several advantages including biocompatibility, bioactivity, and functionality as a scaffold. Here, we found differential structural composition, proteolytic downregulation, and degradation dynamics amongst three ECM grafts. Using *an in vitro* degradation chronic wound model, we found that crosslinked SIS-derived matrices resisted rapid degradation and maintained their functionality as a cellular scaffold through the degradation process. Together, this work highlights key characteristics of native ECM grafts that may predict graft durability and functionality, potentially impacting graft efficacy in supporting wound healing clinically.

## Data Availability

The data used to support the findings of this study are available from the corresponding author upon reasonable request.
